# Diagnostic Accuracy of Artificial Intelligence vs. Oncologist Interpretation in Digital Cervicography for Abnormal Cervical Cytology

**DOI:** 10.3390/jcm14051763

**Published:** 2025-03-06

**Authors:** Kyeong-A So, Eun-Bi Jang, Seung-Hyuk Shim, Sun-Joo Lee, Tae-Jin Kim

**Affiliations:** Department of Obstetrics and Gynecology, KonKuk University Hospital, Seoul 05030, Republic of Korea; 20190001@kuh.ac.kr (K.-A.S.); kiteunbi@kuh.ac.kr (E.-B.J.); 20130131@kuh.ac.kr (S.-H.S.); lsj1121@kuh.ac.kr (S.-J.L.)

**Keywords:** cervicography, artificial intelligence, cervical intraepithelial neoplasia, cervical cancer

## Abstract

**Objective:** We compared the diagnostic performance of artificial intelligence (AI) with that of a gynecologic oncologist during digital cervicography. **Methods:** Women with abnormal cytology who underwent cervicography between January 2019 and December 2023 were included. A gynecologic oncologist interpreted the digital cervicography and the results were compared with those of the AI system. Diagnostic performances were assessed using sensitivity, specificity, positive predictive value (PPV), negative predictive value (NPV), and diagnostic accuracy for low-grade squamous intraepithelial lesions (LSILs) and high-grade squamous intraepithelial lesions (HSILs)/cancer. Cohen’s kappa quantified agreement. **Results:** This study included 449 women (mean age, 41.0 years). A Cohen’s kappa of 0.511 (*p* < 0.0001) indicated moderate agreement between the oncologist and AI. Among 226 cases of HSILs/cancer, the oncologist’s sensitivity was 62.8%, compared to 47.8% for AI, with similar specificity (81.2% vs. 83.5%). The oncologist’s PPV and NPV were 85.0% and 56.3%, respectively, whereas AI’s were 83.1% and 48.5%, respectively. For LSILs/HSILs/cancer (n = 283), the oncologist achieved 98.2% sensitivity and 44.7% specificity, compared to AI’s 93.3% sensitivity and 46.1% specificity. Both had a similar PPV (86.9% vs. 86.6%); however, the oncologist’s NPV (87.2%) exceeded AI’s 64.8%. Diagnostic accuracy for LSILs/HSILs/cancer was 86.9% for the oncologist and 82.3% for AI, whereas for HSILs/cancer, it was 69.6% and 61.0%, respectively. **Conclusions:** Moderate agreement was observed between the oncologist and AI. Although AI demonstrated similar performance in diagnosing cervical lesions, the oncologist achieved higher diagnostic accuracy. AI is a complementary tool and future research should refine AI algorithms to align with clinical performance.

## 1. Introduction

Cervical cancer remains a significant global health challenge, particularly in low- and middle-income countries (LMICs), where it accounts for a large proportion of cancer-related morbidity and mortality among women [1]. According to the World Health Organization (WHO), approximately 94% of 350,000 deaths caused by cervical cancer will occur in LMICs by 2022 [2]. These regions suffer from high cervical cancer incidence and mortality due to a lack of access to essential services, such as human papillomavirus (HPV) vaccination, cervical screening, and timely treatment. The early detection and treatment of cervical lesions are crucial for reducing the burden of cervical cancer. However, limited access to skilled healthcare providers, such as colposcopists, and the subjective nature of visual inspection techniques for identifying cervical lesions pose challenges to accurate diagnosis [3,4]. In resource-limited settings, visual inspection with acetic acid (VIA) is the most common screening method. It is relatively simple to use, inexpensive, and allows for screening and treatment in one visit [5]. VIA is highly subjective, as it relies entirely on healthcare providers’ training and experience. Several studies have suggested high intra- and inter-observer variability, with sensitivities ranging from 25.0% to 94.4% for VIA [6,7,8].

Digital cervicography, a promising tool to address these barriers, is a non-invasive imaging modality for capturing high-resolution images of the cervix after the application of acetic acid, enabling the identification of abnormalities indicative of cervical intraepithelial neoplasia (CIN) and cervical cancer. It is especially advantageous in LMICs because of its cost-effectiveness and ability to standardize assessments compared to the traditional VIA [9,10]. In clinical practice, many countries with a high burden of cervical cancer face a lack of skilled copolscopist [11]. In such settings, cervicography may play a crucial role in enhancing early detection and screening efforts, providing a valuable alternative or complement to traditional biopsy-based methods.

With advancements in technology, artificial intelligence (AI) has shown promise for improving diagnostic capabilities. AI models utilizing deep learning and convolutional neural networks have been increasingly applied in cervical cancer screening, demonstrating promising results for the detection of CIN and cervical cancer [12].

Despite these advancements, questions remain regarding the variability in AI diagnostic performance compared with that of experienced clinicians. To assess the reliability and clinical applicability of AI, agreement between AI interpretations and those by expert gynecologic oncologists is required. This study aimed to evaluate the diagnostic performance of AI in the interpretation of digital cervicography images in patients with abnormal cervical cytology. By analyzing the sensitivity, specificity, and diagnostic concordance between an AI system and a well-trained gynecologic oncologist, this study evaluated the potential role of AI in enhancing cervical cancer screening programs.

## 2. Materials and Methods

This retrospective study included women with abnormal cervical cytology who underwent cervicography-directed biopsies between January 2019 and December 2023. The study was approved by the Institutional Review Board (No. KUMC 2024-10-006). In this method, the cervix is visualized with a vaginal speculum and 5% acetic acid is applied to the cervix. An image of the acetic acid-treated cervix is captured [13]. A well-trained gynecologic oncologist with over 30 years of colposcopic experience reviewed the digital cervicography images and the interpretations were compared with those of an AI system. Both the oncologist and the AI system were blinded to each other’s results throughout the study to ensure unbiased assessment and to minimize potential influence on the outcomes. The cervical image classification AI used was CerviCARE^®^ AI (version 1.1.1; NTL Healthcare Co., Seoul, Republic of Korea). CerviCARE^®^ AI analyzes cervical images to identify cervical regions and classifies them into four categories: negative, atypical, low-grade lesions, and high-grade lesions. In addition, the AI highlights areas with potential lesions to assist medical professionals in making diagnostic decisions. CerviCARE^®^ AI is based on the pre-trained YOLOv10 model and performs transfer learning by adjusting the final output layer using 85,893 cervigrams [14]. The CerviCARE AI was indeed trained on an independent dataset prior to evaluation [11]. For primary validation, the sensitivity was 98.0% and specificity was 95.5%. For secondary validation, CerviCARE AI achieved a sensitivity of 97.5% and a specificity of 95.5%. The positive predictive value (PPV) was 95.6% and the negative predictive value (NPV) was 97.4%.

Cervical images are classified into negative and atypical for normal results and low-grade and high-grade for lesions. Low-grade squamous intraepithelial lesions suggest mild abnormalities in the cervix and are often associated with HPV infection. High-grade squamous intraepithelial lesions indicate more severe abnormalities and are associated with a higher risk of progression to precancerous or cancerous conditions. Diagnostic performance metrics—sensitivity, specificity, positive predictive value (PPV), and negative predictive value (NPV)—were calculated for both methods in detecting low-grade squamous intraepithelial lesions (LSILs) and high-grade squamous intraepithelial lesions (HSILs)/cancer by the pathologic results of cervicography-directed biopsy or cone biopsy. The level of agreement between the oncologist’s and AI’s interpretations was quantified using Cohen’s kappa coefficient. Statistical analyses were performed to assess the diagnostic accuracies of the two methods. A *p* < 0.05 in the two-sided test was considered statistically significant. Statistical analyses were performed using IBM SPSS software (version 21.0; SPSS Inc., Chicago, IL, USA).

## 3. Results

A total of 449 women were included in the study and the diagnostic performances of a well-trained gynecologic oncologist and AI in interpreting digital cervicography images were evaluated.

### 3.1. Interpretation Agreement

An overall Cohen’s kappa of 0.511 (*p* < 0.0001) indicated moderate agreement between the oncologist’s and AI’s interpretations. Table 1 summarizes the comparisons between the oncologist’s and AI’s interpretations. The oncologist and the AI agreed on 85 negative, 106 low-grade, and 110 high-grade lesion/cancer cases. The AI-mismatched negative cases from the oncologist were atypical (six cases), low-grade lesions (eighteen cases), and high-grade lesions/cancers (one case); all atypical cases were incorrectly classified by AI as either a low-grade lesion (eight cases) or negative (one case). The AI’s classification of low-grade lesions compared to the oncologist’s diagnosis resulted in 65 cases being misclassified as high-grade lesions/cancers and 12 as negative. While most high-grade lesion/cancer cases were correctly identified, twenty-five were misclassified as low-grade lesions, two as negative, and one as atypical.

### 3.2. Sensitivity, Specificity, PPV, and NPV

Table 2 summarizes the diagnostic performance of both oncologists and AI in detecting LSILs/HSILs/cancer and HSILs/cancer by comparing the sensitivity, specificity, PPV, and NPV. The oncologist demonstrated higher sensitivity than AI. For LSILs/HSILs/cancer, the oncologist achieved a sensitivity of 98.2%, compared with 93.3% for AI. For HSILs/cancer, the oncologist’s sensitivity was 62.8%, whereas that for AI was 47.8%. In terms of specificity, AI slightly outperformed the oncologist. For LSILs/HSILs/cancer, the AI’s specificity was 46.1%, compared with 44.7% for the oncologist. For HSILs/cancer, the AI’s specificity was 83.5%, while that of the oncologist was 81.2%. The PPV was similar between the two methods, with the oncologist achieving 86.9% for LSILs/HSILs/cancer and 85.0% for HSILs/cancer, whereas the AI achieved 86.6% for LSILs/HSILs/cancer and 83.1% for HSILs/cancer. The NPV was notably higher for the oncologist (87.2% for LSILs/HSILs/cancer and 56.3% for HSILs/cancer) than for AI (64.8% for LSILs/HSILs/cancer and 48.5% for HSILs/cancer).

### 3.3. Diagnostic Accuracy

Table 3 compares the accuracy of the AI and oncologist across the three categories: LSILs, HSILs/cancer, and LSILs/HSILs/cancer. In HSILs/cancer, the oncologist performed significantly better, with an accuracy of 69.6% compared to AI’s 61.0%. In the LSIL category, the oncologist had slightly higher accuracy (61.0%) than the AI (58.80%). For LSILs/HSILs/cancer, the oncologist outperformed AI with an accuracy of 86.9%, compared to 82.3% for AI. 

## 4. Discussion

This study evaluated the diagnostic performance of an AI system in interpreting digital cervicography compared with that of a well-trained gynecologic oncologist. The results showed moderate agreement between the interpretations by the oncologist and the AI in diagnosing cervical lesions. Although the oncologist exhibited superior diagnostic sensitivity and accuracy, AI performed similarly in the diagnosis of cervical lesions. The lower sensitivity of AI compared to that of an experienced oncologist may present challenges, but its comparable specificity and PPV could make it a promising tool for enhancing diagnostic capacity in settings with limited access to specialist care. The higher specificity of AI could help reduce false positives in the detection of cervical lesions. These results suggest that integrating advanced AI with clinical expertise may optimize the screening and diagnosis of cervical cancer, particularly in areas with limited access to experts.

The WHO’s strategy to eliminate cervical cancer includes improving HPV vaccination and expanding screening programs. Recent advancements in the management of cervical disease have been significantly influenced by early diagnosis, surgical techniques, and medical therapies. A notable development is the potential role of HPV vaccination in preventing HPV-related lesions after hysterectomy for high-grade cervical intraepithelial neoplasia (CIN2+) and early-stage cervical cancer. A recent study presented that nonavalent vaccination could potentially cover 94.8% of cases of lower genital tract dysplasia [15]. However, achieving this goal requires overcoming limits in infrastructure, training, and resources, particularly in LMICs [16]. A major advantage of digital cervicography is its ability to provide high-resolution images that can be standardized, compared, and analyzed remotely, which is particularly beneficial in resource-constrained environments. It replaces traditional 35 mm slides with digital images, reducing both the cost and time required to interpret abnormal cervical findings. In Korea, digital cervicography is commonly used in private clinics to examine women with abnormal cytology, particularly when doctors have limited colposcopy experience and require objective documentation of cervical diseases [13]. Cervical biopsies are also performed on patients with abnormal cervicographic findings following expert recommendations. Using high-resolution digital cameras and web-based software, digital cervicography addresses the low reproducibility of photographic assessments [13]. A study evaluating digital cervicography and colposcopy showed that digital cervicography may provide an alternative to colposcopy for diagnosing CIN [17]. Digital cervicography had a higher sensitivity (52.5%) and PPV (60%) than colposcopy (35% and 48.28%, respectively) with a similar specificity (91.8% vs. 91.2%), NPV (89.3% vs. 85.8%), and diagnostic accuracy (84.4% vs. 80.7%).

Furthermore, the simplicity, cost-effectiveness, and potential integration of AI-driven analyses make digital cervicography a promising tool for large-scale cervical cancer screening programs, particularly in regions with limited access to trained clinicians. In recent years, AI has been increasingly applied to diagnosing diseases such as skin tumors [18,19] and retinal diseases [20] and has shown considerable promise. AI can recognize images, extract features, learn classifications, and process data using complex algorithms. The application of AI in the screening and diagnosis of cervical cancer is beneficial for addressing human resource limitations [12]. AI-based tools have demonstrated their utility in automating diagnostic decisions, which could reduce the reliance on highly trained specialists and expand the reach of cervical cancer screening programs.

In this study, sensitivity, specificity, PPV, and NPV analyses provided a comprehensive assessment of the diagnostic performance of digital cervicography with AI, which has shown promising results. Although the sensitivity of AI was lower than that of the oncologist, it was still quite high, especially for detecting LSILs/HSILs/cancer, with a sensitivity of 93.3%. A high sensitivity is critical for cervical cancer screening because it minimizes the risk of false negatives and ensures that cases of cervical precancer or cancer are not overlooked. In addition, the AI interpretation showed slightly better specificity for LSILs/HSILs/cancer, although the difference was marginal (46.1% for AI vs. 44.7% for the oncologist). A higher specificity is desirable to reduce false positives, thus preventing unnecessary treatments and follow-up procedures. These results are similar to those of a previous study grading colposcopic impressions using an AI system, which was shown to have a sensitivity of 90.5% and specificity of 51.8% for diseases graded low-grade or more [21]. The comparable PPVs suggest that both methods are nearly equally effective in predicting true positives. However, the oncologist had a notably higher NPV. This indicates that oncologists are more reliable in correctly identifying true negatives. The lower NPV of AI compared with that of the oncologist (64.8% vs. 87.2% for LSILs/HSILs/cancer) suggests that while AI performs well in identifying lesions, it is not as reliable in ruling out their absence. This finding is a challenge faced by AI, particularly in the reduction of false negatives for high-grade lesions or cancers. Despite advancements in AI technology, these hurdles emphasize the current need for hybrid systems that combine AI with expert human interpretation to enhance diagnostic reliability. AI slightly outperformed the oncologist in specificity for HSILs/cancer (83.5% vs. 81.2%), which suggests that the AI model was better at correctly identifying cases without HSILs, potentially leading to fewer false positives. Clinically, this could imply that while the AI may reduce unnecessary follow-up procedures by minimizing false positives, careful monitoring and additional validation may be needed to ensure that no true cases of HSILs are missed. The lower NPV for AI compared to the oncologist (48.5% vs. 56.3% for HSILs/cancer) suggests that the AI model may have more difficulty accurately ruling out disease. This implies that when the AI classifies a case as negative, there is a higher likelihood of a false negative result compared to the oncologist’s assessment. Clinically, this could lead to missed diagnoses, where patients with HSILs may not be identified for further evaluation. It is important to consider this limitation in the interpretation of AI’s performance. Future research and model refinement are needed to improve the AI’s ability to rule out disease and potentially enhance its overall reliability for clinical use.

In this study, the AI performs reasonably well in detecting LSIL and HSIL/cancer cases, although its accuracy is slightly lower than that of the oncologist. These findings show the potential of AI to enhance cervical cancer screening, particularly in large-scale or resource-limited settings where access to expert oncologists may be limited. However, AI has a relatively low accuracy in detecting HSILs and cancer. Therefore, careful consideration is needed when integrating it into clinical practice. AI may be valuable in triaging cases for further investigation, seeing use as a secondary screening tool with subsequent confirmation by oncologists. AI can assist by rapidly processing and analyzing large volumes of data, enabling the identification of potential cases that require further attention. This capability allows oncologists to focus on more complex cases marked by the AI system for additional review, ultimately improving the efficiency and effectiveness of screening. An AI–oncologist hybrid approach could enhance both sensitivity and specificity in cervical cancer detection. AI can help increase sensitivity by identifying subtle patterns or lesions that may be missed in manual examinations, thus improving the detection of early-stage or low-grade lesions. On the other hand, oncologists bring expertise in interpreting clinical data, which is crucial for accurately diagnosing high-grade lesions and cancer, where AI may have a lower sensitivity or higher rates of false negatives.

It is important to carefully select patients when applying cervicography. Although patients with cervical stenosis were not included in the study, cervical stenosis may present significant challenges in cervicography interpretation. It can limit access to the transformation zone, where high-grade lesions frequently develop [22,23]. This restriction may result in suboptimal acetic acid application, leading to inadequate lesion visualization and an increased risk of false-negative results, particularly in postmenopausal women or those with prior cervical procedures. AI models trained on cervicographic images assume optimal visibility of the cervix, but the presence of cervical stenosis can introduce variability in image quality or the detectability of lesions. Understanding how cervical stenosis impacts the performance of AI compared to expert oncologists could provide valuable insights into the limitations and strengths of AI-assisted cervicography in real-world clinical settings.

Recent advancements in AI have further enhanced the utility of digital cervicography, enabling more consistent and accurate diagnosis of CIN and early-stage cancers. AI algorithms have shown substantial potential in recognizing patterns in cervicographic images, achieving accuracies of approximately 72.1–89% in previous studies [10,24]. Integrating AI with existing digital health interventions, such as smartphone-based VIA systems, offers significant potential for improving accessibility and diagnostic accuracy in resource-limited settings [9]. Moreover, integrating AI with established diagnostic modalities, such as HPV testing and cytology, may further enhance comprehensive cervical cancer screening programs [25]. The continued development and training of AI models on larger and more diverse datasets will enhance their diagnostic performance. Ultimately, a hybrid approach combining AI and expert oncologists may continue to offer the most reliable and accurate diagnostic outcomes.

This study provides valuable insights into the comparative performance of AI and oncologists; however, it has several limitations. First, the AI model’s performance may improve with access to more diverse and comprehensive datasets, as its current capabilities are constrained by the scope of its training data. Second, although the oncologist in this study was well-trained, variability in human interpretation is an inherent limitation that cannot be eliminated. Finally, AI performance could be improved by incorporating more advanced techniques, which could enhance its ability to interpret more complex cases.

## 5. Conclusions

Although AI demonstrates promise for enhancing the specificity and efficiency of the screening and diagnosis of cervical cancer, its current sensitivity limitations warrant further development. Combining AI with clinical expertise can optimize cervical cancer diagnostics, particularly in resource-limited settings. Future research should focus on refining AI algorithms, expanding training datasets, and exploring multimodal diagnostic approaches to establish a more robust and equitable screening framework.

## Figures and Tables

**Table 1:** Agreement between oncologist and AI interpretations of cervical lesions on digital cervicography.

Category		Oncologist				Total
		Negative	Atypical	Low-Grade Lesion	High-Grade Lesion/ Cancer	
AI	Negative	85	6	18	1	110
	Atypical	1	0	8	0	9
	Low-grade lesion	12	9	106	65	192
	High-grade lesion/ Cancer	2	1	25	110	138
Total		100	16	157	176	449
Cohen’s kappa		0.511 (*p* < 0.0001)				

AI, artificial intelligence.

**Table 2:** Comparison of the oncologist’s and AI’s interpretations of digital cervicography in identifying cervical lesions: sensitivity, specificity, PPV, and NPV.

Category		Oncologist	AI
HSILs/Cancer (n = 226)	Sensitivity	62.8%	47.8%
	Specificity	81.2%	83.5%
	PPV	85.0%	83.1%
	NPV	56.3%	48.5%
LSILs/HSILs/Cancer (n = 283)	Sensitivity	98.2%	93.3%
	Specificity	44.7%	46.1%
	PPV	86.9%	86.6%
	NPV	87.2%	64.8%

AI, artificial intelligence; LSILs, low-grade squamous intraepithelial lesions; HSILs, high-grade squamous intraepithelial lesions; PPV, positive predictive value; NPV, negative predictive value.

**Table 3:** Diagnostic accuracy of AI and oncologists’ interpretation of digital cervicography in identifying cervical lesions.

Category	AI Accuracy	Oncologist Accuracy
LSILs	58.80%	61.0%
HSILs/Cancer	61.0%	69.6%
LSILs/HSILs/Cancer	82.3%	86.9%

AI, artificial intelligence; LSILs, low-grade squamous intraepithelial lesions; HSILs, high-grade squamous intraepithelial lesions.

## Data Availability

All the studies used in this study are published in the literature.

## Abbreviations

The following abbreviations are used in this manuscript:

LMIC	low- and middle-income countries
WHO	World Health Organization
HPV	human papillomavirus
VIA	visual inspection with acetic acid
CIN	cervical intraepithelial neoplasia
AI	artificial intelligence
PPV	positive predictive value
NPV	negative predictive value
LSIL	low-grade squamous intraepithelial lesion
HSIL	high-grade squamous intraepithelial lesion
